# Challenges and Solutions to Supporting Physical Literacy within Youth Sport

**DOI:** 10.1007/s40279-025-02313-3

**Published:** 2025-09-17

**Authors:** Kevin Till, Sergio Lara-Bercial, Joseph Baker, David Morley

**Affiliations:** 1 Centre for Child and Adolescent Physical Literacy (CAPL), Carnegie School of Sport, Leeds Beckett University, Leeds, UK; 2 Tanenbaum Institute for Science in Sport, Faculty of Kinesiology and Physical Education, University of Toronto, Toronto, Canada

## Abstract

There are current global concerns surrounding the lifestyle behaviours and future health and well-being of youth. One concept that has gained traction to address these concerns is physical literacy (PL). Organised youth sport is one context that can promote PL, offering multiple benefits coupled with a range of challenges. This Leading Article aims to provide a balanced overview of the key challenges associated with supporting PL within youth sport and offers solutions to overcome these challenges. The first challenge focuses upon attracting youth (and parents) to sport through increasing recruitment against social constraints (e.g., socioeconomic), popular entertainment (e.g., streaming) and family issues (e.g., scheduling). The second centres on retaining children in sport to maximise participation through the appropriate design, organisation and delivery of training and competition opportunities. The final challenge relates to the talent pathway and how sports can structure (e.g., [de]selection) and deliver (e.g., training intensification) a pathway to ensure that all youth athletes flourish along their PL journey. Our solutions focus on organisations (e.g., national governing bodies, clubs) understanding and considering, (1) PL as an individual’s relationship with movement and physical activity throughout life, (2) children’s rights (e.g., interests, opportunities, expression of views), and (3) sport policies and practices when designing and delivering sport experiences. Whilst these challenges and solutions are wide ranging and complex, our belief is that the adoption of a PL approach by stakeholders when designing, delivering and enacting sport programs can enhance the experiences of youth involved in sport and ultimately support their lifelong PL journey.

## Key Points

**Table:** 

Organised youth sport offers a key context to support physical literacy (i.e., our relationship with movement and physical activity throughout life). However, there are challenges to supporting physical literacy in youth sport, including how to (1) attract youth (and parents) to sport, (2) retain youth in sport and (3) structure and deliver talent pathways.
Factors to consider in offering solutions to overcome the challenges associated with youth sport include sports organisation and stakeholders; (1) centralising and prioritising physical literacy, (2) understanding youth’s needs, wants and rights (including the Convention on the Rights of the Child) and (3) the configuration of the sport system.
To achieve this, sports could scale their sport (i.e., modify their competition structure and rules), create new and innovative versions of the sport to minimise dropout, widen their talent pathways, and educate and upskill key stakeholders (e.g., clubs, coaches, parents) to deliver youth sport. However, overcoming these challenges is complex within a multi-layered system but the power of sport can be a positive one when appropriately designed, delivered and enacted.

## Introduction

While the health and well-being of individuals is a primary goal throughout the lifespan, there are current global concerns surrounding the lifestyle behaviours and future health of youth (i.e., children and adolescents) [1]. For example, recent evidence highlights low and decreasing levels of physical activity [2], fitness and motor skill [3] and wellbeing (e.g., increases in anxiety, depression, behavioural problems [4]), alongside increasing obesity levels [5], exacerbated by the COVID-19 pandemic [6]. These health trajectories present a profound challenge to society, resulting from multiple inter-related changes in the current ways of living (e.g., industrialisation [7], technological advances [8, 9], and family structures [10]). As such, there is considerable concern for the short- and long-term future of youth, worldwide. However, limited evidence exists to suggest current interventions or policies are successful in reversing or even decelerating these worrying trends.

One concept that has gained traction over the past two decades in trying to reverse these trends is ‘physical literacy’ (PL; [11, 12]). Since Whitehead’s re-conceptualisation of PL [13], there has been a remarkable worldwide proliferation in the use of the term [14, 15]. Given the all-encompassing nature of Whitehead’s [16] PL definition, as being ‘the motivation, confidence, physical competence, knowledge and understanding to value and take responsibility for engagement in physical activities for life’ it has been employed in several related disciplines (e.g. physical activity, physical education and sport) [15, 17]. Physical literacy has emerged at the forefront of national agendas to generate health benefits for all individuals, but especially for youth [18], showing relationships with physical activity levels [19] and improving academic attainment [20]. Owing to the potential wide-ranging benefits of PL, its importance to youth development is significant. However, concerns have been raised over inconsistent definitions and inappropriate usage of the term [14, 21, 22], and the varying implementation of PL within policy and practice [15, 23].

Subsequently, several nations (i.e., Australia [24], Canada [25], Ireland [26], England [27]) have established PL definitions and/or frameworks to allow the concept to be effectively promoted, supported and enacted in practice. Most recently, Sport England defined PL as ‘our relationship with movement and physical activity throughout life’ [28] based on a co-development phase including 60 experts and 50 organisations [29]. Movement and physical activity were used as an umbrella term encompassing ‘a wide range of activities that involve movement, including but not limited to sport, active recreation, play, exercise, lifestyle activities and active transport.’ Sport England’s PL definition has three main foci: 1) Relationship; having a positive and meaningful relationship with movement and physical activity, but acknowledging this is complex and ever changing; 2) Movement and physical activity; reflecting that individuals move (physical), think (cognitive), feel (affective) and connect (social) during movement and physical activity consistent with previous PL work [30]; and 3) Throughout life, that PL is a lifelong journey influenced by individual, social (e.g., people, communities) and environmental (e.g., spaces, places) factors [31]. Whilst Sport England’s PL definition focusses on all populations (i.e., throughout life), it is important for youth to establish meaningful lifestyle behaviours for adulthood [22, 32] and a lifelong PL journey. Recent research has shown low levels of PL in youth [33, 34] with differences evident based on ethnicity [34], disability [35] and existing medical conditions [36].

One context which could support and enact PL is organised youth sport (i.e., sport that is affiliated to a governing body, is led and managed by adults and typically involves a training and competition schedule). Organised sport is one of the most popular pastimes amongst youth [37], and can promote multiple positive outcomes (e.g., health, academic, wellbeing, personal development), which have been advocated for the last 20 years [38]. However, whilst organised youth sport may offer multiple benefits, there are challenges to promoting PL through the current attraction, retention and talent pathways that sports organisations design and deliver. Organised youth sport can often be delivered through a contested set of beliefs, values and practices that present different challenges and opportunities to PL. For example, the quantity and quality of youth sport engagement must be considered for supporting PL [31, 39]. Whilst previous reviews have presented the challenges associated with related constructs in youth sport (e.g., long-term athletic development [40], talent identification and development [41], movement skill development [31]), to the authors’ knowledge, no paper has attempted to summarise the challenges to supporting PL within organised youth sport. Understanding these challenges and considering proposed solutions may help support youth sport organisations and stakeholders to overcome these global challenges.

To this end, this Leading Article aims to provide a balanced overview of the challenges to supporting PL within youth sport. To achieve this, we consider three areas: (1) attracting youth to sport, (2) retaining youth within sport and (3) the role and impact of the talent pathways applied in sport. Sport England’s PL definition will be adopted and central to discussions throughout the article due to its pragmatic framing of PL as ‘our relationship with movement and physical activity throughout life.’ This definition was designed for cross-sector use within England and is being operationalised across multiple contexts, including sport. Moreover, we have focused upon the key challenges and solutions based on our experiences of researching and working within youth sport over a period of 20–30 years (see Sect. 2), respectively. Table 1 presents the key challenges for supporting PL, which are then discussed in the following sections.

**Table 1:** Challenges to supporting physical literacy within youth sport

Theme	How do sports…	Challenges
1. Attracting youth (and parents) to sport	(a) Increase attraction against social constraints?	Cultural and social value of sport(s)
		Geopolitical (e.g., climate)
		Sociopolitical (e.g., civil war, perceived safety)
		Socioeconomic (e.g. cost of living, cost of sport)
		Low and declining motor skills
	(b) Increase attraction over other popular entertainment?	Developing youth’s relationship with sport
		Youth’s relationship with physically inactive technology
	(c) Allow opportunities for families to logistically meet these needs?	Organised and structured sport delivery and pathways
		Competition between sports
		Logistical and family scheduling
2. Retaining youth in sport	(a) Overcome the challenges associated with attraction to sport?	Socioeconomic (e.g. cost of living, cost of sport)
		Maintaining youth’s relationship with sport over other competing demands
		Competition between sports
		Logistical and family scheduling
	(b) Design and organise training and competition?	Organisation (e.g., competition, leagues)
		Rules (e.g., number of players, field size, equipment)
	(c) Present and deliver their training and competition?	Club structure (e.g., resource, facilities, volunteers)
		Coaches’ practice (e.g., philosophy, knowledge, skills)
		Parents’ behaviour and relationships (e.g., expectations, needs)
3. The talent pathway	(a) Structure their talent pathway?	Talent identification and selection policies (e.g., age, number of opportunities)
		Financial and transfer regulations
		Experiences of (non)selected and deselected participants
		Deselection procedures
	(a) Deliver their talent pathway?	Intensification of talent pathways (e.g., training and competition scheduling)
		Negative impacts associated with talent pathways (e.g., injury, pressure, academic attainment)
		Competing demands of youth athletes (e.g., academic, vocational, social)

## Positionality of the Authors

All authors work within universities in the UK, Canada or Australia and have expertise researching and/or working within youth sport. All authors are associated with a research centre related to PL and youth development. For example, K.T., S.L.B. and D.M. are all colleagues within the Centre for Child and Adolescent Physical Literacy at Leeds Beckett University. Furthermore, the authors all have (or have had) an applied role working with youth sport. For example, one author is a parent of 2 children (aged 8 and 12 years), coaches an Under 9s community rugby league team and is a strength and conditioning coach within a talent development setting. As such, the authors acknowledge that their research and applied experiences will have shaped the challenges, solutions, discussions and ideas presented within the paper but believe these first-hand experiences allow the presentation of contextually relevant information resulting in a more in-depth understanding of PL within youth sport [42]. However, it must be acknowledged that these experiences and positionality present bias to the article, including the challenges and solutions presented. For example, the authors’ research and applied experiences within sport pathways (from participation to talent systems) are the main reason that the challenges have been presented in terms of (1) attracting youth to sport, (2) retaining youth within sport and (3) the talent pathway in sport.

## Challenges

### Attracting Youth (and Parents) to Sport

Attraction to sport exists in broad societal and cultural contexts, whereby changes in such contexts have the potential to attract youth to sport in meaningful or inconsequential ways. For example, Baker and Horton [43] emphasized the influence cultural and social factors can have on sport engagement, specifically the increased likelihood of sports participation where sports have high cultural and social value (e.g., ice hockey in Canada, cricket in India, basketball in the USA). Furthermore, Emmonds and colleagues [44] reported significantly greater participation rates in soccer, recognised as a national sport in many European countries, across Europe compared with 17 other sports. In addition, boys were four times more likely to participate in sport compared with girls [44]. As such, the cultural value of sport, the exposure (e.g., TV, media), role models and governmental funding will more than likely impact how youth think about, connect with and feel towards sport, both in general and within specific sports.

Alongside culture, geo-political (e.g., climate change, extreme weather) and socio-political (e.g., civil war, famine, perceived safety) factors likely affect sport attraction [45, 46] and may deter youth (and/or their parents) from pursuing sport. For example, climate change, extreme weather events and the threat (and reality) of war are removing spaces and opportunities to be active due to unsafe environments or extreme conditions [46, 47]. Furthermore, the increasing cost of sport may leave many families unable to fund sports participation, sport clubs and opportunities to coach [48, 49]. This presents socioeconomic inequities [50], but other factors such as sex, ethnicity and disability [51] are also relevant. How can youth develop a meaningful relationship with movement and physical activity if opportunities are not available or the cost is too high?

How children spend their time has also changed, offering additional challenges to attracting youth to sport. For example, streaming services, social media and gaming (although some can be physically active) use have increased and changed the interests of youth [9, 52]. Furthermore, sports activities have become more organized and structured over the last two decades [48] with sports often delivered in silos, focussed on commercialisation. This can result in competition between sports for participants and increasing logistical challenges (e.g., time, scheduling, cost, travel) for parents, often undermining recommendations for multi-sport activity [40]. Furthermore, low and declining motor skills lead to lower competence and confidence within youth, which may affect how they are attracted to sports, especially for girls and younger children [53, 54]. Overall, the paradox is that children commonly play less, instead spending more time in non-physical activities or structured training and competition, than previous generations. This could be compounded by societal influences (e.g., parental concerns that it is not safe for children to play outside by themselves or to use recreational facilities; [55, 56]). Current practices do not appear to align with physical activity guidelines [57] and developmental models for sports participation [58], likely affecting the competence and confidence of children to be attracted to sport. In summary, key questions to attracting youth to sport include how do sports (a) increase attraction through social means (e.g., reducing barriers, increasing opportunities)? (b) increase attraction over other popular entertainment (e.g., gaming)? and (c) allow opportunities for families to logistically meet these needs?

### Retaining Youth in Sport

Once a youth (and parent) is attracted to sport, ensuring regular attendance and retainment within the short- (i.e., weekly), medium- (i.e., seasonal) and long- (i.e., as long as possible) term is key towards achieving a relationship with movement and physical activity ‘for life.’ It is likely that short- and medium-term retention will more likely lead to supporting PL and could lead to lifelong involvement even if not competing in the sport (e.g., recreational activity, coaching). However, research suggests youth sport participation is declining [44, 48], especially during adolescence (i.e., 14–18 years; [44]), questioning the existence and quality of individual positive and meaningful relationships with sport. Explanations for declining participation trends and reduced retention within youth sport have included intrapersonal (e.g., lack of enjoyment), interpersonal (e.g., parent pressure), and structural (e.g., injury) constraints [59] alongside the restrictive sports governance applied by national governing bodies [40, 60, 61]. However, recent research [62] has challenged these reductionist approaches, suggesting sports participation is a highly individualised and multifaceted process with no ‘one size fits all’ solutions.

Sports should consider some of the same challenges (e.g., costs, between sports competition, logistical) for attracting and retaining youth (and parents) within sport to avoid dropout. Recent research in Flanders (Belgium) [61] found sports organisations spent more time on attraction than investing in quality provision to aid retention of their current participants. This has implications for organisations in the investment of resources (e.g., financial, time, staff) to both attract and retain youth to sport. Furthermore, Lara-Bercial and colleagues [62] found that the top three reasons for sport dropout across Europe were that youth 1) prioritised school and had no time for sports, 2) found it stressful if they did not perform well and 3) found other activities to do that they enjoyed more than sport. These findings suggest some factors outside of a sport’s control may impact attraction and retainment, highlighting the challenges for prioritising sport in society.

Whilst sporting organisations may not be able to control all variables affecting an individual’s attraction to sport and the quality of their participation, delivering fun, enjoyable and appropriate activities have been identified as the main reasons youth participate in sport [63, 64]. Therefore, two further challenges to retention include how youth sport is a) designed and b) delivered. National governing bodies create policies for the design and organisation of youth sport to provide direction and influence practice [65], which are vital for establishing guidelines for delivery by stakeholders [66]. For example, scaling sport (or competitive engineering [67]), is a design method that involves manipulating the organisation (e.g., competition) and rules (e.g., field size, number of players, equipment) of a sport to determine the behaviours, actions and skills performed [66]. This is not a new concept, with research suggesting scaling sport leads to more skill opportunities, success, engagement and self-efficacy [66], likely resulting in more meaningful PL. Whilst scaling youth sport is commonly applied across multiple sports (e.g., Gaelic games [68], rugby league [67]), whether this results in an environment conducive to youth flourishing on their PL journey could be questioned on the basis of recent participation trends [44, 59]. However, limited research is available that evaluates these sport modifications from a participation and PL perspective (e.g., [68, 69]).

Whilst national organisations can create policies, sports clubs and coaches enact, implement and deliver them [68, 70]. National organisations have a responsibility to ensure coaches are appropriately trained and ready to deliver suitable training and competition for their participants, which is a challenge in itself due to the complexity of coaching [71]. Owing to the nature of PL, and the multiple motivations of youth for sports participation, meeting the varying needs of all children [72] to retain them in sport is seen as more complex than performance coaching [73]. Furthermore, parental involvement (and their needs) can make this even more complex [74]. Therefore, delivering sport practices to support PL and retain children in sport on a short-, medium- and long-term basis is challenging. Whether many youth sport clubs have the infrastructure and resource, and coaches have the philosophy, knowledge and skills to achieve this could be questionable [75].

### The Talent Pathway

Whilst national sport organisations have a sports participation agenda, there is also a strong drive to develop future elite and/or professional athletes [41]. As such, talent pathways have become popular in youth sport [76]. Talent pathways refer to the talent identification and development systems and structures offered within sports, typically employing a pyramidal structure, whereby the number of places available on the pathway decreases as the support provided (e.g., higher qualified coaches, contact-time, facilities, services) increases at progressive levels based upon the resources available [41]. Talent pathways commence at different ages in different sports (e.g., 7 years in soccer [77]; 15 years in rugby union [78]), and have been the subject of much discussion [79, 80]. However, from a PL perspective, the talent pathway may influence an individual’s relationship with movement and physical activity owing to the (non)opportunities it provides. Till and Baker [41] previously discussed the challenges associated with talent pathways, focusing on how the talent pathway is structured and delivered, which may have implications for PL [81].

The structure of a talent pathway results in limited opportunities for youth athletes to be identified and selected into a talent programme (e.g., approx. 15 places in a football academy [77]). Furthermore, these opportunities can come with financial and transfer regulations [82]. Whilst there is considerable research on talent identification within youth sport (e.g., [83–85]), limited research (e.g., [86]) exists exploring youth athletes’ (selected, non-selected, deselected) perceptions and experiences of the talent identification and selection process. Experiences differ for [un]successful individuals, which may have detrimental effects on sports participation and supporting PL for life [87]. Furthermore, deselection from a talent pathway is common (e.g., 29% player turnover per season in soccer academies [88]) with psychological distress a common outcome [89, 90]. Therefore, as much as talent pathways are critiqued from a performance perspective [41, 79, 81], they may also have wider impact upon youth PL. As such the structure, policies and implementation of talent pathways may be a challenge towards PL for youth athletes.

Alongside the structure of talent pathways, concerns have been raised within the academic literature [81, 91, 92] and popular media [93] about the delivery of talent programmes and intensification of youth sport. These concerns range from excessive training loads [94], insufficient rest and recovery [95], injury [96], social factors (e.g., time away from family and friends [81]), pressure [97], eating disorders [98], academic achievement [99] and safety amongst parents [100]. Whilst this research has considered these challenges from a health rather than a PL perspective, most of these concerns could impact a youth’s relationship with sport. Against this negative backdrop, research has nonetheless also demonstrated multiple positive outcomes of engagement in talent pathways (e.g., enhanced fitness, enhanced health, social interaction, relationship building, improved mental health) with recent research demonstrating short- and long-term outcomes related to life skills, educational and vocational opportunities and readiness for life [101–106]. Therefore, a challenge for sport organisations is how to design pathways to maximise the positive and minimise the negative outcomes of sport within the short- and long-term to ensure every individual has a positive relationship with their sport and with physical activity in general. However, this is challenging as youth can often participate in multiple sports simultaneously (e.g., train and compete for multiple teams across several sports [107, 108]) and have other developmental demands (e.g., education, vocation, social [106]). Furthermore, the mutual exclusiveness delivered by some sports can result in some youth not having the opportunity to experience other sports and build a broader base to support PL.

### Summary

A multitude of challenges have been presented and discussed in relation to supporting PL within youth sport. From an ecological perspective, these challenges apply across the macro (i.e., society), meso (i.e., sport organisations) and micro (i.e., individual contexts) levels [109], reflecting the complexity of the challenges. Whilst some solutions were hinted at in the discussion above, the following section will present some general solutions to support overcoming these challenges.

## Solutions

**Fig. 1:**
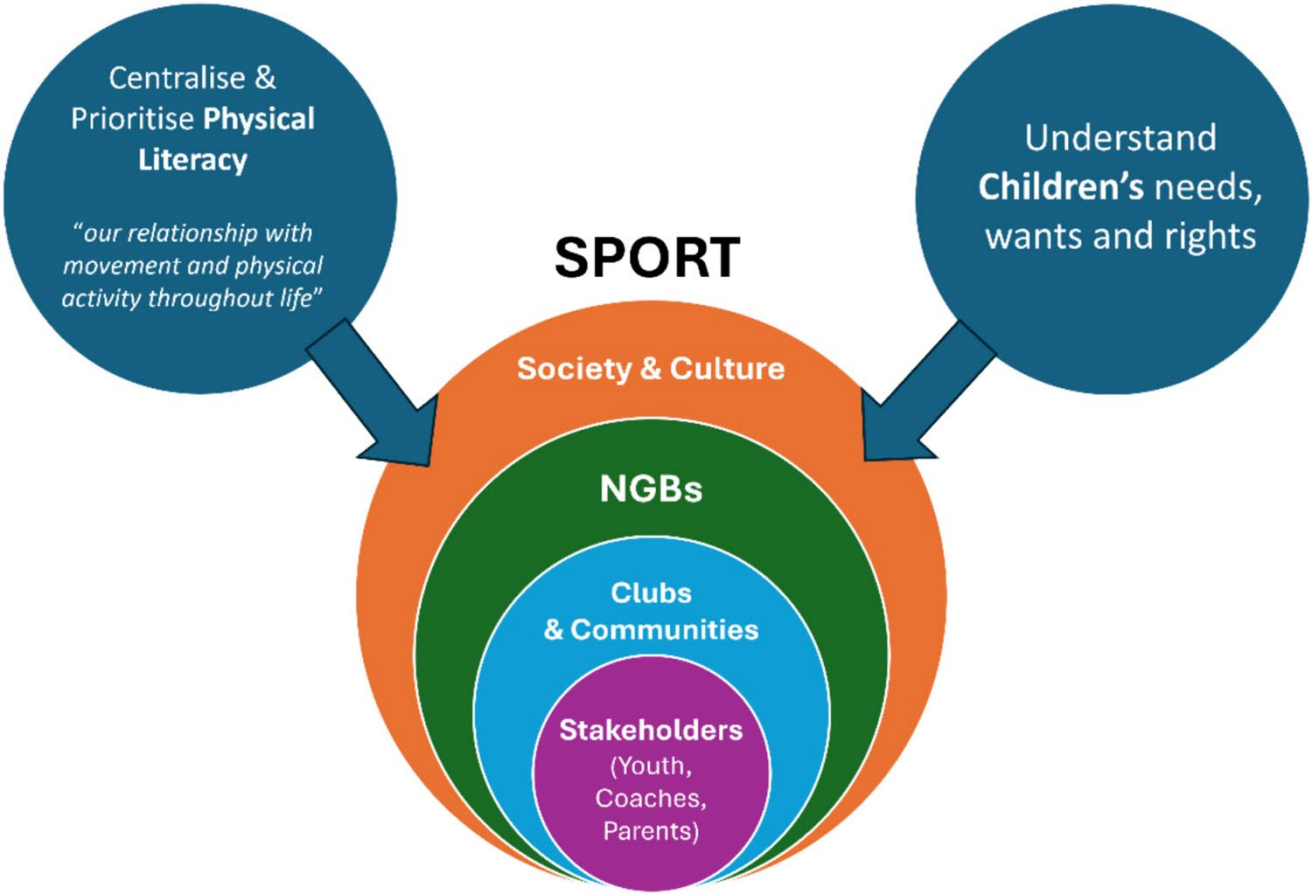
Solutions to support physical literacy in youth sport. Notes: The circles represent the ecological perspective of the levels of youth sport. Society and culture represent how a sport may sit within this but also where other movement sectors may influence youth sport. NGBs, national governing bodies

This paper has focussed on supporting PL within youth sport. Three main factors to consider in offering solutions to overcome the challenges discussed are: (1) centralising and prioritising PL, (2) understanding youth’s needs, wants and rights, and (3) the configuration of the sport system. Figure 1 presents a conceptual overview of these solutions.

### Centralising and Prioritising Physical Literacy

As discussed, PL is a worldwide term at the forefront of national agendas but is often misinterpreted and misused [14, 21, 22]. The establishment of clear PL definitions and frameworks helps offer a solution to sport organisations to apply to their vision and objectives. Our recommendation is that youth sport organisations (e.g., national governing bodies, sport federations, clubs) clarify, centralise and prioritise PL for all participants (e.g., new, existing, different abilities) within their strategies and policies across youth (and adult) sport. Whilst the time for the Sport England PL consensus statement (i.e., ‘our relationship with movement and physical activity throughout life’ [28]) to translate into changes to youth sport has been short (i.e., since 2023), it provides a framework for thinking about PL within youth sport. The three main foci—(1) Relationship (i.e., meaning, value, enjoyment); (2) Sport (i.e., how youth move [physical], think [cognitive], feel [affective], connect [social]); and (3) Throughout Life (i.e., influenced by individual, social, environmental factors)—provide a structure for sports organisations to use to attract and retain participants within their sport alongside developing future talent [110]. Such a solution may help organisations ask appropriate questions whilst understanding the associated benefits of making this part of national strategies. Researchers have advocated embedding PL within the curriculum [111] to support PL education, implementation and enactment [112] whilst others have shown positive emotions leading to enhanced value and motivation in physical education [110]. Furthermore, future research in other contexts and on stakeholders’ understanding of PL is required.

### Youth’s Needs, Wants and Rights

Whilst the PL consensus statement was defined for all populations, its greatest impact may be for youth to establish early meaningful relationships with movement and physical activity that are maintained into adulthood. It is important to acknowledge that ‘children are not mini adults’ and their wants and needs from a PL perspective will differ between individuals, ages and stages (e.g., child versus adolescent). Therefore, understanding youth development from a biopsychosocial developmental perspective would help support the ability to meet their needs and wants [113–115]. A further consideration is that children have a universal right to be protected as per the United Nations Convention on the Rights of the Child (UNCRC; [116]). This convention was published to protect children’s rights and to support governments and organisations to ensure children may flourish in their endeavours. The UNCRC thus offers an additional framework to guide PL in sport. Whilst 54 UNCRC articles exist, six key rights should be strongly considered from a PL and youth sport perspective (Table 2), regardless of whether these actions stem from governments, sport organisations or individuals. Recent initiatives (e.g., Play Their Way [117], ICOACHKIDS Putting Kids first in sport [118]) have been developed to promote child-first practice on the basis of the rights of the child. Such considerations within youth sport may encourage stronger relationships to be developed between children and sport.

**Table 2:** United Nations Convention on the Rights of the Child appropriate to Physical Literacy

Article no	Summary	Article	Implications
2	Non-discrimination	The Convention applies to every child without discrimination, whatever their ethnicity, sex, religion, language, abilities or any other status, whatever they think or say, whatever their family background	Organisations delivering youth sport should consider how they create opportunities for all youth
3	Best interests of the child	The best interests of the child must be a top priority in all decisions and actions that affect children	Organisations delivering youth sport could include a reference to always having ‘the best interest of the child’ or to being ‘child-centred’ in their policies and mission statements. This will facilitate and simplify decision-making
6	Life, survival and development	Every child has the right to life. Governments must do all they can to ensure that children survive and develop to their full potential	Organisations could create pathways that support more youth. Coaches could deliberately target the development of the children they work with beyond sport and physical skill to incorporate character development and life skills
12	Respect for children’s views	Every child has the right to express their views, feelings and wishes in all matters affecting them, and to have their views considered and taken seriously	Organisations and coaches could put in place a variety of mechanisms to encourage children to form and express their views in anything that matters to them. They could then make changes to the way the organisation and their activities are run on the basis of this feedback
13	Freedom of expression	Every child must be free to express their thoughts and opinions	As above
15	Freedom of association	Every child has the right to meet with other children and to join groups and organisations	Organisations could enhance and facilitate access to their activities for all children. They could also conduct special recruitment drives in hard-to-reach populations
27	Adequate standard of living	Every child has the right to a standard of living that is good enough to meet their physical and social needs and support their development. Governments must help families who cannot afford to provide this	Organisations could create pathways that support more youth. Coaches could deliberately target the development of the children they work with beyond sport and physical skill to incorporate character development and life skills
31	Leisure, play & culture	Every child has the right to relax, play and take part in a wide range of cultural and artistic activities	Organisations and coaches could ensure that they create a climate where children can relax and be playful and where they do not experience unnecessary stress or pressure during their activities

### The Sport System

There is no doubt that organised youth sport is a contributor to PL. Centralising and prioritising PL and understanding youth needs, wants and right could allow the sport system (from society and culture to stakeholders) to work towards supporting PL within youth sport. To begin, sport organisations can review and evaluate existing policies and then design and implement new ones aligned to maximising relationships with the sport and how different children (e.g., new, existing, different abilities) may move, think, feel and connect with their sport. To attract youth (and parents), sports need to understand current problems to attraction (e.g., cost, other forms of entertainment). For example, learning how to better engage youth, and encouraging them to be active, potentially through the use of technology, may offer a powerful tool to ‘nudge’ youths into being more physically active [119, 120].

To attract and retain youth, sports organisations should consider the scaling of their sport across different ages and stages. For example, research has demonstrated positive implementation of scaled competitions and rules across multiple sports [67, 121, 122]. Table 3 presents a series of questions that sports could consider when scaling their sport for youth. Whilst there are no right or wrong answers, there are many available options to scale sports to help maximise the move, think, feel and connect components of PL. Aligning participant enjoyment, a positive climate and the biopsychosocial wants, needs and rights of youth would seem appropriate. Opportunities to do this that are supported by peers (play), are cost-effective, and do not require high levels of commitment may increase uptake. Likewise, sports could work to create new and innovative versions of the game which encourage and allow more youth, especially during adolescence, to remain engaged with the sport in more informal ways (e.g., 3 × 3 basketball [123]; 5-a-side football [124]). Evaluating the experiences of those involved in scaled sport through the intended-enacted-experienced curriculum model [68] may help inform such designs and implementation. Furthermore, organisations (e.g., clubs, schools) could consider how they offer multi-sport, movement and physical activity opportunities for youth that allows individuals to experience multiple activities and families to manage logistical challenges.

**Table 3:** Considerations for Scaling the Organisation and Rules of Youth Sport Competition

Question for sports organisations	Example answers
1. What factors does your sports competition system emphasise?	Standings and tables, cup competitions, winning, keeping score
2. How are your game schedules organised?	League format vs. multiple seasons within a calendar year vs. festival vs. friendlies
3. How many players can register for a club?	Limited number of registrations, how many?
4. What are the rules implemented?	Number of players per side, playing area size, rule modifications
5. How do you group players?	Age category vs. body size [130, 131] vs. performance. Separated by sex/gender or combined
6. How many children can play at once?	Limited number of players (& substitutes) vs. opportunities for everyone to play

Those working in more competitive sport pathways could stop thinking about talent per se and consider ways to apply appropriate practices to everyone (or as many as possible) for as long as possible [41]. This approach would be an appropriate response to the considerable evidence against early (de)selection in sport. Furthermore, sports should consider how they can work together to minimise logistical challenges and cost whilst providing appropriate places and spaces to undertake sport.

Lastly, sport organisations have a responsibility for educating stakeholders involved in the sport, including clubs, coaches and parents. The PL statement emphasises the communities, people and spaces of youth sport, recognising that stakeholders usually create and influence the environment. Stakeholders have a responsibility to develop movement, feeling, connection and thinking in relation to their activity, which could be viewed as competence, motivation, confidence and knowledge as per previous PL definitions [16]. Coaches could do this within training and competition by using appropriate learning activities and coach behaviours to maximise the elements of PL [125, 126] or by organising training and competition according to the needs, wants and level of the child. Other stakeholders may influence this through co-operation with coaches [127] and their actions and behaviours both on and off the field [128]. Education seems paramount for upskilling stakeholders with examples of free coach education for youth available and examples of best practice [129].

### Limitations

This Leading Article provides an overview of the key challenges associated with supporting PL within youth sport and offers solutions to overcome these challenges. However, it is important to note some limitations. First, the article aimed to offer a balanced overview on the basis of the research literature and the authors’ research and experiences in youth sport, and did not use a systematic approach. Second, the article used the recent Sport England consensus statement, but PL definitions differ between nations. Furthermore, it is important in enhancing experiences for youth that definitional debates do not become additional obstacles to making positive change. Third, owing to biases in the current research landscape, this article generally focused on research from higher socioeconomic status nations and has not collected the broad views of other stakeholders (youth, parents, coaches). Fourth, a range of solutions are offered, and whilst we believe they are conceptually robust, they need testing for feasibility across nations, sports and contexts for implementation within real-world settings. Lastly, whilst organised youth sport is the focus within this article, it must be strongly acknowledged that youth sport is only one context for supporting PL, and other contexts (e.g., active recreation, play, exercise, lifestyle activities, active transport, physical education, performing arts, adult sport, leisure and fitness) may play equal, or more important, roles in enhancing PL throughout the life course.

## Conclusions

PL has been proposed as a way of addressing current concerns about the lifestyle behaviours and future health and well-being of youth globally with recent position statements (e.g., Sport England [28]) emphasising its importance. Organised youth sport is a context to support PL offering many benefits. However, to achieve this, challenges must be understood and overcome. The current paper presents three challenges: namely, the attraction of youth (and parents) to sport, retaining youth within sport and the impact of the talent pathway. Solutions focus on centralising and prioritising PL, understanding youth’s needs, wants and rights, and the sport system applying policies and practices to design and deliver better sport experiences for youth. This is certainly a complex challenge within a multi-layered system of organisations and stakeholders. To achieve this, it is important to translate and disseminate knowledge and current best practices with future research required to understand the challenges and current knowledge of stakeholders delivering on the ground. Our belief is that the power of sport can be a positive one when designed, delivered and enacted through all stakeholders using a PL lens. Only in this way can sport remain relevant and therefore be central to overcoming the global challenges faced by today’s youth.
